# Disorders of the calcium-sensing receptor and partner proteins: insights into the molecular basis of calcium homeostasis

**DOI:** 10.1530/JME-16-0124

**Published:** 2016-10-01

**Authors:** Fadil M Hannan, Valerie N Babinsky, Rajesh V Thakker

**Affiliations:** 1 Academic Endocrine UnitRadcliffe Department of Medicine, University of Oxford, Oxford, UK; 2 Department of Musculoskeletal BiologyInstitute of Ageing and Chronic Disease, University of Liverpool, Liverpool, UK

**Keywords:** signal transduction, parathyroid and bone, kidney, G protein

## Abstract

The extracellular calcium (Ca^2+^_o_)-sensing receptor (CaSR) is a family C G protein-coupled receptor, which detects alterations in Ca^2+^_o_ concentrations and modulates parathyroid hormone secretion and urinary calcium excretion. The central role of the CaSR in Ca^2+^_o_ homeostasis has been highlighted by the identification of mutations affecting the *CASR* gene on chromosome 3q21.1. Loss-of-function *CASR* mutations cause familial hypocalciuric hypercalcaemia (FHH), whereas gain-of-function mutations lead to autosomal dominant hypocalcaemia (ADH). However, *CASR* mutations are only detected in ≤70% of FHH and ADH cases, referred to as FHH type 1 and ADH type 1, respectively, and studies in other FHH and ADH kindreds have revealed these disorders to be genetically heterogeneous. Thus, loss- and gain-of-function mutations of the *GNA11* gene on chromosome 19p13.3, which encodes the G-protein α-11 (Gα_11_) subunit, lead to FHH type 2 and ADH type 2, respectively; whilst loss-of-function mutations of *AP2S1* on chromosome 19q13.3, which encodes the adaptor-related protein complex 2 sigma (AP2σ) subunit, cause FHH type 3. These studies have demonstrated Gα_11_ to be a key mediator of downstream CaSR signal transduction, and also revealed a role for AP2σ, which is involved in clathrin-mediated endocytosis, in CaSR signalling and trafficking. Moreover, FHH type 3 has been demonstrated to represent a more severe FHH variant that may lead to symptomatic hypercalcaemia, low bone mineral density and cognitive dysfunction. In addition, calcimimetic and calcilytic drugs, which are positive and negative CaSR allosteric modulators, respectively, have been shown to be of potential benefit for these FHH and ADH disorders.

## Introduction

**Figure 1:**
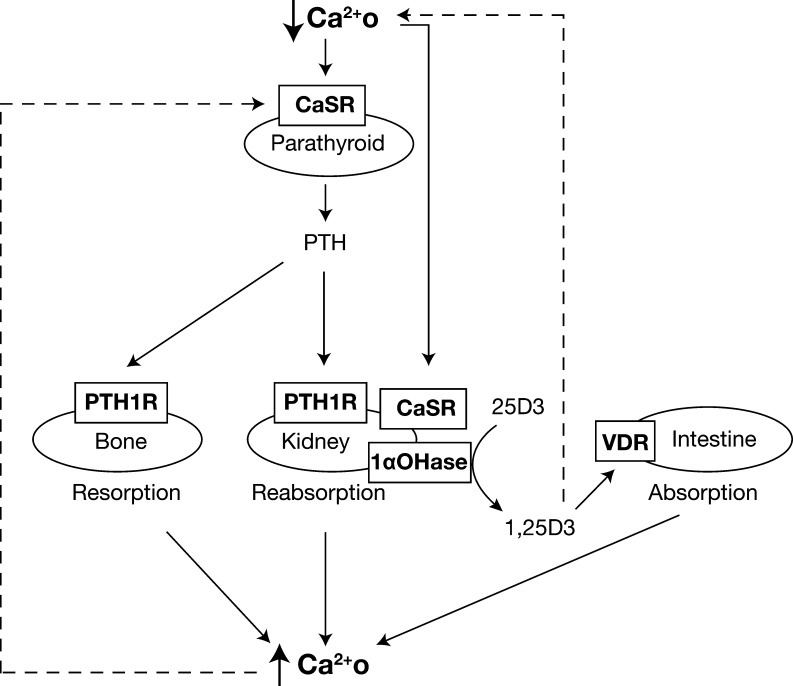
Overview of Ca^2+^_o_ homeostasis. The parathyroid CaSR senses reductions in Ca^2+^_o_, which leads to a rapid rise in PTH secretion. The increased circulating PTH acts via the PTH1-receptor (PTH1R) in the kidneys and bone. The skeletal effects of PTH are to increase bone resorption, thereby releasing calcium into the extracellular fluid. In the kidney, PTH increases calcium reabsorption and stimulates the proximal renal tubular 1-α-hydroxylase (1αOHase) enzyme, which promotes the synthesis of the active 1,25-dihydroxyvitamin D3 (1,25D3) metabolite from 25-hydroxyvitamin D3 (25D3), which is the major circulating form of vitamin D. The elevated 1,25D3 acts on the intestine via the vitamin D receptor (VDR) to increase the absorption of dietary calcium. Thus, in response to hypocalcaemia, the secretion of PTH, by these direct and indirect actions leads to the restoration of normocalcaemia. The kidney CaSR senses reductions in Ca^2+^_o_ and promotes urinary calcium reabsorption independent of the actions of PTH. The rise in Ca^2+^_o_ and 1,25D3 concentrations mediated by PTH act on the parathyroid glands to induce feedback inhibition of further PTH secretion.

Extracellular calcium (Ca^2+^_o_) is essential for the regulation of a variety of biological processes including neuromuscular excitability, blood coagulation and mineralisation of bone matrix and for providing a steady supply of calcium for intracellular functions ranging from muscle contraction to hormone synthesis and secretion (Brown 1991). Thus, there is a requirement for Ca^2+^_o_ to be tightly regulated, and a complex homeostatic system has evolved to maintain Ca^2+^_o_ at near-constant concentrations (Fig. 1) (Thakker 2016). The Ca^2+^_o_ homeostatic system comprises four main components: (1) the parathyroid glands, which sense the prevailing Ca^2+^_o_ concentrations and regulate the actions of target calcitropic organs; (2) the intestine and kidneys, which facilitate the transfer of calcium between the external environment and extracellular fluid; (3) the skeleton, which represents the major reservoir of total body calcium and is critical for buffering short-term changes in Ca^2+^_o_ concentrations; and (4) calcitropic hormones such as parathyroid hormone (PTH) and 1,25-dihydroxyvitamin D_3_, which mediate the interactions between the parathyroid glands, bone, kidney and intestines (Fig. 1). Thus, low Ca^2+^_o_ concentrations lead to PTH release from the parathyroid glands. This hormone exerts three distinct effects on Ca^2+^_o_ homeostasis, which are to enhance bone resorption, urinary calcium reabsorption, and the renal synthesis of 1,25-dihydroxyvitamin D_3_, thereby leading to intestinal calcium absorption (Fig. 1). Low Ca^2+^_o_ concentrations are also sensed by the kidneys, which increase urinary calcium reabsorption independent of the actions of PTH (Loupy* et al*. 2012, Riccardi & Valenti 2016). These combined events mediate a rise in Ca^2+^_o_ concentrations, which together with 1,25-dihydroxyvitamin D_3_, leads to feedback inhibition of PTH secretion (Fig. 1) (Thakker 2016).

Studies of patients and kindreds with familial hypocalciuric hypercalcaemia (FHH) or autosomal dominant hypocalcaemia (ADH) have helped to elucidate the molecular basis of Ca^2+^_o_ sensing and overall regulation of Ca^2+^_o_ homeostasis by calcitropic tissues such as the parathyroid glands and kidneys. These studies also revealed the involvement of a G protein-coupled receptor (GPCR) pathway that comprises the calcium-sensing receptor (CaSR), G-protein α-11 (Gα_11_) subunit and adaptor-related protein complex 2 sigma (AP2σ) subunit (Pollak* et al*. 1993, Nesbit* et al*. 2013*a*,*b*). This article will provide an overview of the role of the CaSR, Gα_11_ and AP2σ proteins, review the disorders caused by mutations of these proteins, outline potential targeted therapies, and describe insights gained into the molecular basis of Ca^2+^_o_ homeostasis by studies of the CaSR and its partner proteins.

## Overview of calcium-sensing receptor signalling and trafficking

**Figure 2:**
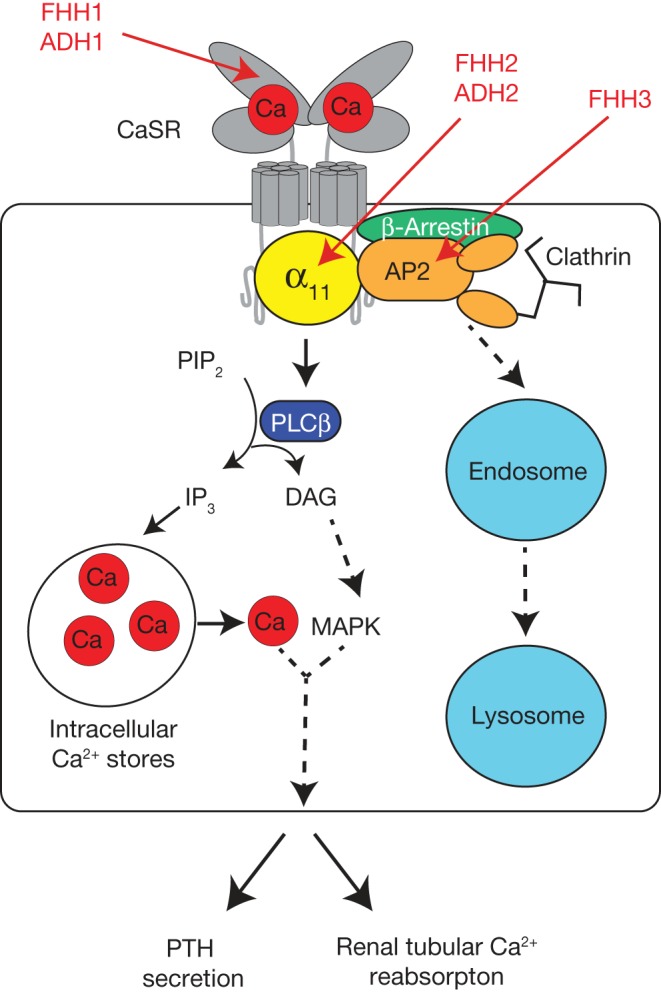
Role of the CaSR, Gα_11_ and AP2 complex in the regulation of PTH secretion and renal tubular calcium reabsorption. The binding of calcium (red filled-in circle, Ca) to the extracellular bilobed venus fly trap (VFT) domain of the CaSR (grey) results in Gα_11_ (yellow)-dependent stimulation of phospholipase C-β (PLCβ) (dark blue) activity, which catalyses the formation of inositol 1,4,5-trisphosphate (IP_3_) and diacylglycerol (DAG) from phosphatidylinositol 4,5-bisphosphate (PIP_2_). An accumulation of IP_3_ mediates the rapid release of calcium into the cytosol from intracellular stores, whereas DAG activates the MAPK cascade. These intracellular signalling events lead to a decrease in PTH secretion from the parathyroid chief cell and reduction in renal tubular calcium reabsorption. CaSR cellsurface expression is regulated by agonist-driven insertional signalling (ADIS) (not shown) (Grant *et al*. 2011) and also by an endocytic complex comprising clathrin, β-arrestin (green) and the AP2 complex (orange), which traffic this GPCR to the endosomal-lysosomal degradation pathway (light blue) or recycle the CaSR back to the cell surface (Breitwieser 2013). Loss- and gain-of-function mutations of the CaSR lead to FHH1 and ADH1, respectively, whereas loss- and gain-of function mutations of the Gα_11_-subunit are associated with FHH2 and ADH2, respectively. Loss-offunction mutations of the AP2σ-subunit lead to FHH3.

The human CaSR is a 1078 amino acid dimeric cell-surface protein that belongs to class C of the GPCR superfamily, which also includes the metabotropic glutamate, gamma-aminobutyric acid type B (GABA_B_) and taste 1 receptors (Katritch* et al*. 2013). The CaSR is highly expressed in the parathyroid glands and kidneys (Regard* et al*. 2008). It has a large (612 amino acid) extracellular domain (ECD), which was recently crystallised and shown to comprise two globular lobes that adopt a venus flytrap (VFT) conformation (Zhang* et al*. 2016). The cleft region located between the two lobes of the VFT is predicted to bind Ca^2+^_o_ (Fig. 2) (Huang* et al*. 2007, Hannan* et al*. 2012), thereby leading to the activation of multiple intracellular signalling cascades via interactions with its transmembrane and intracellular domains (Conigrave & Ward 2013, Hofer & Brown 2003). In the parathyroid glands, the G_q/11_ family of heterotrimeric guanine nucleotide–binding proteins (G proteins) are considered to represent the major downstream signalling partners for the CaSR (Varrault* et al*. 1995, Wettschureck* et al*. 2007, Nesbit *et al*. 2013*a*). The binding of the CaSR to the G_q_ and G_11_ proteins is predicted to lead to the dissociation of the respective Gα-subunits from their obligate Gβγ heterodimer, which activate the phospholipase C-beta (PLCβ) enzyme, thereby facilitating the hydrolysis of a plasma membrane lipid constituent, known as phosphatidylinositol 4,5-bisphosphate (PIP_2_), to produce inositol 1,4,5-trisphosphate (IP_3_) and diacylglycerol (DAG) (Fig. 2) (Hofer & Brown 2003, Conigrave & Ward 2013). IP_3_ in turn stimulates the rapid release of calcium from intracellular stores (Breitwieser & Gama 2001), whereas DAG activates the mitogen-activated protein kinase (MAPK) cascade (Corbetta* et al*. 2002). These intracellular events mediate a decrease in PTH secretion from the parathyroid chief cell and a reduction in renal tubular calcium reabsorption (Fig. 2). The level of CaSR cell-surface expression likely influences Ca^2+^_o_ homeostasis and is regulated by agonist-driven insertional signalling (ADIS), which leads to enhanced anterograde trafficking of newly synthesised receptors to the plasma membrane after prolonged exposure to Ca^2+^_o_ (Grant* et al*. 2011). Moreover, the β-arrestin protein together with the adaptor-related protein complex 2 (AP2), which comprises a heterotetrameric complex of α-, β-, μ- and σ-subunits (Nesbit *et al*. 2013*b*) is considered to facilitate CaSR internalisation from the plasma membrane by clathrin-mediated endocytosis and retrograde trafficking (Breitwieser 2013). Thus, AP2 and β-arrestin likely repre­sent key determinants of CaSR cell-surface expression.

## Disorders associated with calcium-sensing receptor mutations

**Figure 3:**
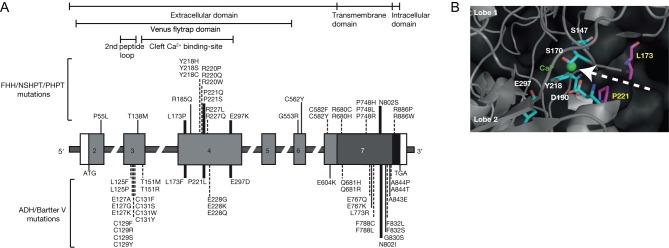
(A) Schematic representation of the genomic organisation of the *CASR* gene showing mutational hotspots for disease-associated missense mutations. The *CASR* gene consists of six coding exons (2–7), and the start (ATG) and stop (TGA) codons are in exons 2 and 7, respectively. The 5′ portion of exon 2 and the 3′ portion of exon 7 are untranslated (open boxes). The 3′ portion of exon 2, exons 3, 4, 5 and 6 and the 5′ portion of exon 7, encode the extracellular domain (light grey). The mid portion of exon 7 encodes the transmembrane (dark grey) and intracellular (black) domains. More than 170 different missense *CASR* mutations have been reported in patients with FHH1, NSHPT, adult-onset PHPT, ADH1 and Bartter syndrome type V (Hannan & Thakker 2013). These mutations affect >110 different codons scattered throughout the *CASR* gene. Twenty-eight codons (>20% of all mutated codons) represent mutational hotspots as they are the site of recurrent missense mutations (solid lines) that have been reported in three or more probands (mutational frequency of >1.5%), and/or affected by multiple (3 or more) different missense mutations (dashed lines). Mutations affecting these codons are clustered in three regions, which are the 2nd peptide loop of the extracellular domain, venus flytrap (VFT) cleft region Ca^2+^_o_ binding site, and the region encompassing transmembrane domains (TMD) 6 and 7. Codons 173, 221, 297 and 802 (thickened lines) are the site of missense mutations that may lead to a loss- or gain-of-function and are termed ‘switch’ codons or residues. (B) Homology modelling of the CaSR VFT cleft region ligand-bound Ca^2+^_o_ binding site based on the crystal structure of the metabotropic glutamate receptor type 1. The Leu173 (L173) and Pro221 (P221) switch residues are predicted to be located in short α-helices within lobes 1 and 2, respectively, that form the entrance to the Ca^2+^_o_ binding site within the VFT cleft. The L173 and P221 side chains are shown in purple, the side chains of predicted Ca^2+^_o_ binding residues (Ser147 (S147), Ser170 (S170), Asp190 (D190), Tyr218 (Y218) and Glu297 (E297)) are shown in cyan, and a bound calcium ion is shown as a green sphere. The side chains of L173 and P221 are predicted to extend across the entrance to the Ca^2+^_o_ binding site. Mutations affecting these residues may lead to opposing effects on CaSR function by influencing the entry and binding of calcium within the VFT cleft region. Adapted, with permission, from Hannan FM, Nesbit MA, Zhang C, Cranston T, Curley AJ, Harding B, Fratter C, Rust N, Christie PT, Turner JJ, *et al. *(2012) Identification of 70 calcium-sensing receptor mutations in hyper- and hypo-calcaemic patients: evidence for clustering of extracellular domain mutations at calcium-binding sites. *Human Molecular Genetics* **21** 2768–2778. Copyright 2012 Oxford University Press.

More than 230 different germline mutations of the CaSR, which is encoded by the *CASR* gene (Fig. 3) located on chromosome 3q21.1, have been reported (Hannan & Thakker 2013). These mutations may cause a loss of CaSR function and give rise to hypercalcaemic disorders such as FHH type 1 (FHH1), neonatal severe hyperparathyroidism (NSHPT) and adult-onset primary hyperparathyroidism (PHPT); or lead to a gain of function that is associated with hypocalcaemic disorders such as ADH type 1 (ADH1) and Bartter syndrome type V (Table 1) (Hannan & Thakker 2013).

**Table 1:** Familial disorders of Ca^2+^_o_ sensing.

**Disorder**	**OMIM**	**Inheritance**	**Gene**	**Chromosomal localisation**	**Clinical features**
Hypercalcaemic disorders					
Familial hypocalciuric hypercalcaemia type 1 (FHH1)	145980	Autosomal dominant	*CASR*	3q21.1	Asymptomatic
Familial hypocalciuric hypercalcaemia type 2 (FHH2)	145981	Autosomal dominant	*GNA11*	19p13.3	Asymptomatic
Familial hypocalciuric hypercalcaemia type 3 (FHH3)	600740	Autosomal dominant	*AP2S1*	19q13.3	Hypercalcaemic symptoms in >20% of cases Low bone mineral density in >50% of cases Childhood cognitive deficits in >75% of cases
Neonatal severe hyperparathyroidism (NSHPT)	239200	Autosomal recessive or dominant	*CASR*	3q21.1	Hyperparathyroid bone disease Hypercalcaemic symptoms
Adult-onset primary hyperparathyroidism (PHPT)	–	Autosomal recessive or dominant	*CASR*	3q21.1	Nephrolithiasis in >40% of cases Low bone mineral density in >25% of cases
Hypocalcaemic disorders					
Autosomal dominant hypocalcaemia type 1 (ADH1)	601198	Autosomal dominant	*CASR*	3q21.1	Hypocalcaemic symptoms in ~50% of cases Ectopic calcifications in ~35% of cases
Autosomal dominant hypocalcaemia type 2 (ADH2)	615361	Autosomal dominant	*GNA11*	19p13.3	Hypocalcaemic symptoms in >75% of cases Short stature reported in a single kindred
Bartter syndrome type V	601198	Autosomal dominant	*CASR*	3q21.1	Renal salt wasting and hypokalaemia Hypocalcaemic symptoms in >75% of cases

### Familial hypocalciuric hypercalcaemia type 1 (FHH1)

FHH comprises three genetically distinct conditions, designated as FHH types 1–3 (Table 1), which are due to loss-of-function mutations affecting the CaSR, Gα_11_ and AP2σ proteins, respectively (Pollak *et al*. 1993, Nesbit *et al*. 2013*a*,*b*). FHH1 (OMIM #145980) is the most common type and accounts for ~65% of all FHH cases (Hannan *et al*. 2012). FHH is a highly penetrant autosomal dominant condition characterised by lifelong non-progressive mild-to-moderate hypercalcaemia, normal (in ~80% of patients) or mildly raised serum PTH levels (in ~20% of patients) (Firek* et al*. 1991), and low urinary calcium excretion (in ~95% of patients) (Marx 2015). FHH is considered to be a benign disorder, as patients are typically asymptomatic (Hannan & Thakker 2013). However, an increased prevalence of chondrocalcinosis with advancing age (Volpe* et al*. 2009) and occasional cases of pancreatitis have been reported (Pearce* et al*. 1996*c*). Individuals with FHH have been misdiagnosed as having PHPT and undergone parathyroidectomy, which generally fails to normalise the hypercalcaemia (Hannan & Thakker 2013). Mutational analysis may be required to distinguish FHH from PHPT, and to date, FHH1 has been associated with >130 different mutations of the *CASR* gene (Fig. 3), which are missense substitutions in >85% of cases, whereas nonsense, deletion, insertion and splice-site mutations that lead to truncated CaSR proteins have been described in <15% of cases (Hannan *et al*. 2012, Hannan & Thakker 2013). Studies of FHH1-associated CaSR mutations have identified critical receptor structure–function relationships and demonstrated a mutational hotspot within the ECD. Indeed, an analysis of the locations of recurrent FHH1-causing CaSR mutations or residues affected by multiple different loss-of-function CaSR mutations has revealed a clustering of mutations at the major predicted Ca^2+^_o_ binding site located within the cleft region of the bilobed extracellular VFT domain of the CaSR (Fig. 3) (Hannan *et al*. 2012). These mutated residues may be directly involved in the binding of Ca^2+^_o_ or indirectly influence alterations in receptor conformational states that occur upon Ca^2+^_o_ binding (Huang *et al*. 2007, 2009, Hannan *et al*. 2012). Moreover, *in vitro* functional expression studies have identified specific VFT domain residues, which when mutated can result in opposing effects on CaSR responses and lead to a loss or gain of function (Fig. 3) (Hannan *et al*. 2012, Zhang* et al*. 2014). These residues may potentially act as intra-molecular switches to regulate the entry and binding of Ca^2+^_o_ within the VFT cleft region (Fig. 3) (Hannan *et al*. 2012, Zhang *et al*. 2014). FHH-causing mutations also cluster in the CaSR transmembrane domain (TMD) and may inhibit the transmission of activation signals to the intracellular environment, by impairing interactions with heterotrimeric G proteins and other components of the CaSR signal transduction pathway (Leach* et al*. 2012). Some loss-of-function CaSR mutations have been shown to cause signalling bias by switching the wild-type CaSR from preferentially coupling with intracellular Ca^2+^ (Ca^2+^_i_) to a mutant receptor that signals equally via the Ca^2+^_i_ and MAPK pathways, or which predominantly acts via MAPK (Leach *et al*. 2012). Loss-of-function CaSR mutations causing signalling bias are located within the ECD and TMD; however, the structural motifs within these regions that determine whether the CaSR preferentially couples to Ca^2+^_i_ or MAPK pathways remain to be established. Around 50% of CaSR mutations that lead to FHH1 have been shown to result in reduced cell-surface receptor expression as a consequence of defective trafficking to the plasma membrane, with mutant CaSRs being retained intracellularly and unable to exit either the endoplasmic reticulum or Golgi apparatus (Huang & Breitwieser 2007, White* et al*. 2009). Such cellular studies also provide an explanation for the benign phenotype of FHH1, which is a heterozygous condition, by demonstrating that co-expression of wild-type and mutant FHH1-causing CaSRs ameliorates the loss of function, with the co-expressed wild-type CaSRs increasing trafficking of mutant receptors to the plasma membrane via the ADIS mechanism (Grant* et al*. 2012). Whereas, cells expressing only mutant loss-of-function CaSRs have both defective membrane targeting and reduced signalling responses (Grant *et al*. 2012).

### Neonatal severe hyperparathyroidism (NSHPT)

NSHPT (OMIM #239200) is a potentially life-threatening disorder most often caused by homozygous or compound heterozygous loss-of-function CaSR mutations (Table 1) (Chattopadhyay & Brown 2006, Hannan *et al*. 2012, Hannan & Thakker 2013). NSHPT is characterised by severe neonatal hypercalcaemia (serum calcium concentrations are typically 3.5–5.0 mmol/L), 5- to 10-fold elevations of serum PTH concentrations, marked parathyroid gland enlargement, failure to thrive, and hyperparathyroid skeletal disease leading to multiple fractures and respiratory distress (Hannan & Thakker 2013, Murphy* et al*. 2016). Parathyroidectomy is usually required to treat NSHPT, and bisphosphonates have been successfully used in some patients to manage marked hypercalcaemia and skeletal demineralisation before parathyroid surgery (Waller* et al*. 2004, Wilhelm-Bals* et al*. 2012). Severe neonatal hypercalcaemia is also reported to be associated with heterozygous loss-of-function CaSR mutations, and these findings indicate that NSHPT may be due to factors other than mutant gene dosage. For example, the degree of severity of a dominant-negative mutation or maternal serum calcium concentration may play a role in the phenotypic expression of a CaSR mutation in the neonate (Hannan & Thakker 2013). More than 25 different CaSR mutations have been described in association with NSHPT, of which >40% are either nonsense or frameshift mutations that are predicted to lead to a truncated CaSR (Hannan *et al*. 2012, Hannan & Thakker 2013).

### Primary hyperparathyroidism (PHPT) and marked hypercalcaemia presenting after infancy

Loss-of-function CaSR mutations may occasionally present after the neonatal period with marked hyper­calcaemia. Indeed, homozygous loss-of function mutations, which are located at the N-terminal region of the CaSR, have been reported to lead to symptomatic hypercalcaemia in childhood or early adulthood, which required treatment with parathyroidectomy (Chikatsu* et al*. 1999, Miyashiro* et al*. 2004). Occasionally, heterozygous and homozygous loss-of function CaSR mutations have been detected in adult patients with PHPT caused by parathyroid adenomas or hyperplasia (Table 1) (Hannan* et al*. 2010*a*). The occurrence of PHPT or severe FHH after infancy may be due to the degree of loss of function associated with the underlying CaSR mutations. Indeed, the homozygous CaSR mutations, present in these patients, have been associated with milder alterations in Ca^2+^_i_ signalling than homozygous muta­tions leading to NSHPT (Hannan *et al*. 2010*a*). An analysis of CaSR mutations identified in patients with PHPT or severe FHH that presented after infancy has indicated that CaSR mutations located in the receptor ECD are associated with more severe hypercalcaemia. This hypercalcaemia is recalcitrant to treatment by parathyroidectomy, possibly due to multiple gland involvement (Hannan *et al*. 2010*a*). These findings may have some clinical utility in identifying those patients (i.e. with CaSR ECD mutations) in whom a full neck exploration is required, as they are more likely to have multi-gland disease (Hannan *et al*. 2010*a*, Hannan & Thakker 2013).

### Autosomal dominant hypocalcaemia type 1 (ADH1) and Bartter syndrome type V

ADH comprises two genetically distinct disorders, designated as ADH types 1 and 2 (Table 1), which are caused by germline gain-of-function mutations of the CaSR and Gα_11_ proteins, respectively (Pollak* et al*. 1994, Pearce* et al*. 1996*b*, Nesbit *et al*. 2013*a*). ADH1 (OMIM #601198) accounts for ~70% of all ADH cases (Nesbit *et al*. 2013*a*). ADH is characterised by mild-to-moderate hypocalcaemia, with serum calcium concentrations, adjusted for variations in serum albumin concentrations, rarely below 1.50 mmol/L, and ~50% of patients may develop hypocalcaemic symptoms such as paraesthesia, carpopedal spasms and seizures (Pearce *et al*. 1996*b*, Hannan & Thakker 2013, Nesbit *et al*. 2013*a*). Although ADH is associated with increased circulating phosphate concentrations and inappropriately low or normal PTH concentrations, this is considered to represent a distinct disease entity from hypoparathyroidism. This is because affected individuals generally have PTH concentrations that are detectable and within the reference range and also a relative hypercalciuria that is characterised by urinary calcium-to-creatinine ratios that are within or above the reference range (Pearce *et al*. 1996*b*, Hannan & Thakker 2013, Nesbit *et al*. 2013*a*). Ectopic calcification of the kidneys and basal ganglia is a common feature of ADH and affects >35% of patients (Pearce *et al*. 1996*b*, Hannan & Thakker 2013, Nesbit *et al*. 2013*a*). Patients with severe gain-of-function CaSR mutations may also have Bartter syndrome type 5, which is characterised by hypokalaemic alkalosis, renal salt wasting and hyperreninaemic hyperaldosteronism (Vargas-Poussou* et al*. 2002, Watanabe* et al*. 2002, Kinoshita* et al*. 2014). Mutational analysis is commonly required for the diagnosis of ADH, and >70 different CaSR mutations have been identified to date in individuals affected with ADH1 (Hannan *et al*. 2012, Hannan & Thakker 2013). Of these mutations, 95% are heterozygous missense substitutions (Hannan & Thakker 2013). The structure–function analyses of these mutations have defined key peptide motifs of the CaSR that maintain this receptor in an inactive conformation. In particular, gain-of-function mutations cluster within the second peptide loop of the extracellular VFT domain (residues 116–136) (Fig. 3) (Jensen* et al*. 2000) that contributes to the interface of the dimeric CaSR (Hu & Spiegel 2007, Zhang *et al*. 2016). Mutations affecting this extracellular peptide loop may lead to a gain of function by promoting conformational changes such as dimer rotation that facilitates receptor activation (Hu & Spiegel 2007). A second hotspot for ADH1-associated mutations is located in a region that encompasses transmembrane domains 6 and 7, and the intervening third extracellular loop of the CaSR (residues 819–837) (Fig. 3) (Hu* et al*. 2005). This region is likely important for locking the ligand-unbound family C GPCRs in an inactive conformation by forming a network of interactions with other transmembrane domains (Dore* et al*. 2014) that inhibits the binding of G proteins. Cellular studies have demonstrated that most ADH1-causing CaSR mutations cause a signalling bias by coupling more strongly to Ca^2+^_i_ mobilisation than to the MAPK pathway, which contrasts with FHH1-causing CaSR mutations, which are biased towards MAPK signalling (Leach *et al*. 2012).

### Use of calcimimetic and calcilytic drugs for disorders caused by CaSR mutations

Calcimimetics are ligands that mimic or enhance the effects of Ca^2+^_o_ at the CaSR and are divided into two types: type I calcimimetics are agonists, which include naturally occurring ligands such as polyvalent cations; and type II calcimimetics are positive allosteric modulators that include l-amino acids and cinacalcet, which is a synthetic amino alcohol compound (Nemeth* et al*. 2004). Calcilytics are inhibitors of CaSR function, and to date, no endogenous calcilytic compounds have been identified. However, synthetic calcilytic compounds have been generated and shown to act as negative allosteric modulators of the CaSR (Nemeth & Shoback 2013). A recent study has identified a putative binding cavity for synthetic calcimimetic and calcilytic compounds within the extracellular portion of the CaSR TMD and delineated key glutamate residues within the binding cavity, which may facilitate the conformational changes that lead to altered receptor activity upon the binding of these compounds (Leach* et al*. 2016). Calcimimetic drugs represent a potential targeted therapy for NSHPT and symptomatic forms of FHH1. They are shown to improve the signalling by loss-of-function CaSR mutants *in vitro* (Rus* et al*. 2008, Leach* et al*. 2013) and to act as pharmacochaperones that facilitate correct protein folding and plasma membrane targeting of mutant CaSRs (Huang & Breitwieser 2007, White *et al*. 2009). These ﻿*in vitro* effects of calcimimetic drugs are in keeping with reports of FHH1 patients with marked hypercalcaemia or complications such as recurrent pancreatitis who have responded to treatment with cinacalcet, which is licensed for the management of certain hyperparathyroid disorders (Timmers* et al*. 2006, Festen-Spanjer* et al*. 2008). Cinacalcet has also been successfully used to manage life-threatening hypercalcaemia in NSHPT probands harbouring a heterozygous CaSR mutation, Arg185Gln (Gannon* et al*. 2014). However, it is ineffective for NSHPT caused by biallelic deletional CaSR mutations (Atay* et al*. 2014).

Symptomatic ADH is conventionally treated with calcium and active vitamin D preparations. However, these therapies do not rectify the underlying pathophysiological alterations in parathyroid and renal tubular function, and their use predisposes affected individuals to the development of marked hypercalciuria, nephrocalcinosis, nephrolithiasis and renal impairment (Pearce *et al*. 1996*b*, Hannan & Thakker 2013, Nesbit *et al*. 2013*a*). ADH1 patients have also been treated with recombinant PTH (1–34) (teriparatide); however, when administered by once- or twice-daily bolus injections, this peptide may not always prevent hypercalciuric renal complications (Theman* et al*. 2009). Calcilytic drugs, which are CaSR negative allosteric modulators, represent a potential targeted therapy for ADH1. Calcilytics comprise two main classes of compounds, which are the amino alcohols (e.g. NPS-2143, ronacaleret and JTT-305/MK-5442) and quinazolinones (e.g. ATF936 and AXT914) (Nemeth & Goodman 2016). *In vitro* studies have shown that NPS-2143, a long-acting calcilytic, corrects the gain of function associated with ADH-causing CaSR mutations (Hu *et al*. 2005, Letz* et al*. 2010, Lia-Baldini* et al*. 2013, Hannan* et al*. 2015*b*). However, the *in vitro* efficacy of NPS-2143 was reduced by mutations affecting NPS-2143-binding residues within the TMD (Hu *et al*. 2005, Letz *et al*. 2010). In contrast, the quinazolinone calcilytic drugs (ATF936 and AXT914) have been demonstrated to rectify the excessive signalling responses of all ADH mutants evaluated to date, including those mutations leading to constitutive activation and/or Bartter syndrome type 5 (Letz* et al*. 2014). To assess whether calcilytics may ameliorate the hypocalcaemia associated with ADH1, these drugs have been administered to mouse models harbouring germline gain-of-function CaSR mutations. In a single-dose *in vivo* study, NPS-2143 was administered to *Nuf* mice, which have hypocalcaemia, reduced plasma PTH concentrations and ectopic calcification in association with a germline gain of function Casr mutation, Leu723Gln (Hough* et al*. 2004, Hannan *et al*. 2015*b*). Intraperitoneal injection of NPS-2143 significantly increased plasma calcium and PTH concentrations in heterozygous- and homozygous-affected *Nuf* mice at 1 h after administration, with values returning to baseline after 4 h. The elevations in plasma calcium induced by NPS-2143 were not associated with any increase in urinary calcium excretion (Hannan *et al*. 2015*b*). Longer-term *in vivo* studies involving the JTT-305/MK-5442 calcilytic compound have been undertaken in two ADH1 mouse models, which harbour germline Cys129Ser and Ala843Glu gain-of-function CaSR mutations, respectively (Dong* et al*. 2015). Administration of JTT-305/MK-5442 by daily oral gavage over a 12-week period led to sustained increases in serum calcium concentrations and a significant reduction in urinary calcium excretion in both ADH1 mouse mutants (Dong *et al*. 2015). Recently, a calcilytic compound known as NPSP795 has been evaluated in a clinical trial involving five ADH1 patients. The intravenous administration of NPSP795 significantly increased plasma PTH concentrations and reduced urinary calcium excretion (Ramnitz* et al*. 2015). However, circulating calcium levels were not altered in this study. The optimal dosing regimen for NPSP795, which is a short-acting calcilytic compound intended to elicit a rapid and transient increase in plasma levels of PTH, remains to be established in ADH1 patients (Ramnitz *et al*. 2015).

## Disorders associated with G-protein α-11 subunit mutations

**Figure 4:**
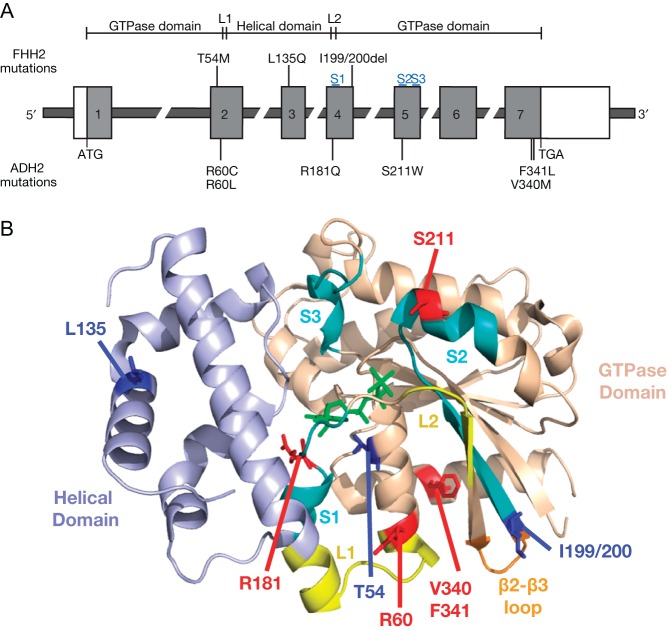
(A) Schematic representation of the genomic organisation of the *GNA11* gene showing germline disease-associated mutations. The *GNA11* gene consists of 7 coding exons (1–7), and the start (ATG) and stop (TGA) codon are in exons 1 and 7, respectively. The 5′ portion of exon 1 and the 3′ portion of exon 7 are untranslated (open boxes). The 3′ portion of exon 2, exon 3 and 5′ portion of exon 4 encode the Gα_11_ helical domain, which is connected by two short peptides, termed linker 1 (L1) and linker 2 (L2), to the GTPase domain. The Gα_11_ GTPase domain is encoded by the 3′ portion of exon 1, 5′ portion of exon 2, 3′ portion of exon 4 and exons 5–7. Three flexible regions, termed switch regions 1–3 (S1–S3, shown in blue), which undergo conformational changes during Gα_11_ activation are encoded by exons 4 and 5. The location of reported FHH2- and ADH2-causing mutations is shown. (B) Three-dimensional homology model of Gα_11_ showing the location of residues mutated in FHH2 (blue) and ADH2 (red). The homology model is based on the crystal structure of Gα_q_ (PDB accession number 3AH8) (Nishimura *et al*. 2010), which shares 90% amino acid identity with Gα_11_. The Gα_11_ helical and GTPase domains are shown bound to GDP aluminium fluoride (GDP-AlF_4_, green), which is a non-hydrolysable analogue of GTP. The three flexible switch regions are highlighted in cyan, and the L1 and L2 peptides are shown in yellow. The β2–β3 hairpin loop, which comprises part of the Gα–GPCR interface, is shown in orange. Adapted, with permission, from Nesbit MA, Hannan FM, Howles SA, Babinsky VN, Head RA, Cranston T, Rust N, Hobbs MR, Heath H 3rd & Thakker RV (2013) Mutations affecting G-protein subunit alpha11 in hypercalcemia and hypocalcemia. *New England Journal of Medicine* **368** 2476–2486. Copyright 2013 Massachusetts Medical Society.

Germline mutations affecting Gα_11_, which is encoded by the *GNA11* gene (Fig. 4) on chromosome 19p13.3, have recently been identified as the genetic cause of FHH and ADH in some patients. This finding has revealed Gα_11_ to be a major component of the CaSR signalling pathway and highlighted its importance in Ca^2+^_o_ homeostasis. Loss-of-function Gα_11_ mutations give rise to FHH2 (Nesbit *et al*. 2013*a*, Gorvin* et al*. 2016), whereas germline gain-of-function Gα_11_ mutations are associated with ADH2 (Fig. 4 and Table 1) (Mannstadt* et al*. 2013, Nesbit *et al*. 2013*a*, Li* et al*. 2014, Piret* et al*. 2016) and somatic gain-of-function Gα_11_ mutations cause uveal melanomas (Van Raamsdonk* et al*. 2010).

### Familial hypocalciuric hypercalcaemia type 2 (FHH2)

The existence of a genetically distinct form of FHH was first highlighted by genetic linkage studies that mapped an *FHH* locus to chromosome 19p, which contains the *GNA11* locus. This form of FHH was designated as FHH2 (OMIM #145981) (Table 1) (Heath* et al*. 1993). *GNA11* was considered a likely candidate gene for FHH2, as this encodes the Gα_11_ protein, which is highly expressed in the parathyroid glands (Varrault *et al*. 1995) and acts as a signalling partner for the CaSR (Hofer & Brown 2003). Moreover, mouse model studies have demonstrated parathyroid specific ablation of Gα_11_, and the related Gα_q_ protein, to result in marked hypercalcaemia, hyperparathyroidism and parathyroid gland enlargement (Wettschureck *et al*. 2007). DNA sequence analysis of the reported FHH2 kindred revealed a germline heterozygous *GNA11* mutation that resulted in an in-frame isoleucine deletion at codon 199 or 200 (Ile199/200del) of the Gα_11_ protein (Nesbit *et al*. 2013*a*). Mutational analysis of the *GNA11* gene in additional FHH probands, who did not harbour *CASR* mutations, has identified heterozygous Leu135Gln and Thr54Met missense mutations in two unrelated probands (Nesbit *et al*. 2013*a*, Gorvin *et al*. 2016). The Thr54Met, Leu135Gln and Ile199/200del Gα_11_ mutations are associated with a mild FHH phenotype characterised by serum adjusted calcium concentrations <2.80 mmol/L (Nesbit *et al*. 2013*a*, Gorvin *et al*. 2016). In keeping with these clinical findings, *in vitro* studies have shown that these FHH2-associated Gα_11_ mutations lead to a mild impairment of CaSR signal transduction (Nesbit *et al*. 2013*a*, Gorvin *et al*. 2016). Indeed, the Thr54Met, Leu135Gln and Ile199/200del FHH2 mutants were associated with around a 30% increase in the half-maximal effective concentration (EC_50_) of CaSR-expressing cells, whereas CaSR mutations leading to FHH1 generally cause a >50% increase in the EC_50_ value (Bai* et al*. 1996, Pearce* et al*. 1996*a*, Hannan *et al*. 2012).

Homology modelling revealed that FHH2-causing mutations are located within key regions of the Gα_11_-subunit, which consists of a GTPase domain that binds GDP and GTP and a smaller helical domain that acts as a clasp to secure these bound guanine nucleotides (Fig. 4) (Oldham & Hamm 2008). Thus, the Ile199/200del mutation is located within the GTPase domain and predicted to disrupt a hairpin loop, which comprises part of the Gα-GPCR interface and is also situated between flexible ‘switch’ regions (Fig. 4) that undergo substantial conformational changes upon GTP binding (Nesbit *et al*. 2013*a*). In contrast, the Leu135Gln mutation is located in the Gα_11_ helical domain (Fig. 4) (Nesbit *et al*. 2013*a*). It is not predicted to influence CaSR–Gα_11_ coupling, but instead likely diminishes CaSR signal transduction by influencing the interaction of Gα_11_ with downstream effectors. The Thr54Met mutation is located at the interface between GTPase and helical domains (Fig. 4) and predicted to impair coupling and/or dissociation of Gα_11_ from the CaSR by influencing guanine nucleotide binding at the inter-domain interface (Gorvin *et al*. 2016). Thus, the identification of these FHH2-causing Gα_11_ mutations has revealed residues critical for Gα_11_-subunit function.

### Autosomal dominant hypocalcaemia type 2 (ADH2)

After the identification of loss-of-function Gα_11_ mutations leading to FHH2, it was hypothesised that gain-of-function germline Gα_11_ mutations may have opposite effects on Ca^2+^_o_ homeostasis and give rise to a disorder with an ADH-like phenotype. DNA sequence analysis of eight ADH probands, who did not harbour CaSR mutations, identified germline heterozygous Gα_11_ mutations in two individuals (Nesbit *et al*. 2013*a*). *In vitro* functional studies of these mutations, which comprised Arg181Gln and Phe341Leu Gα_11_ missense substitutions, demonstrated cells expressing the mutant Gα_11_ proteins to have enhanced sensitivity to Ca^2+^_o_, consistent with a gain of function (Nesbit *et al*. 2013*a*). In parallel with these studies, genetic linkage studies of two unrelated hypocalcaemic kindreds mapped the disease locus to chromosome 19p13.3, which is the location of the *GNA11* locus. DNA sequence analysis revealed the occurrence of germline heterozygous Gα_11_ mutations, Arg60Cys and Ser211Trp (Mannstadt *et al*. 2013). Moreover, heterozygous Arg60Leu and Val340Met Gα_11_ mutations have been identified by whole-exome sequencing in additional ADH kindreds, and these mutations were demonstrated to lead to enhanced CaSR-mediated signal transduction (Li *et al*. 2014, Piret *et al*. 2016). These individuals and families with gain-of-function Gα_11_ mutations, who were designated as having ADH2 (OMIM #615361) (Table 1), generally had serum-adjusted calcium concentrations ranging 1.75–2.15 mmol/L. The affected individuals typically presented with hypocalcaemic symptoms such as paraesthesia, muscle cramps, carpopedal spasm and seizures (Mannstadt *et al*. 2013, Nesbit *et al*. 2013*a*, Li *et al*. 2014, Piret *et al*. 2016). Some ADH2 patients were susceptible to treatment-related hypercalciuria, nephrocalcinosis and nephrolithiasis (Li *et al*. 2014, Piret *et al*. 2016), although affected individuals generally had a milder urinary phenotype, with significantly reduced urinary calcium excretion compared with ADH1 patients who harbour gain-of-function CaSR mutations (Li *et al*. 2014). Furthermore, patients with germline gain-of-function Gα_11_ mutations, in contrast to patients with gain-of-function CaSR mutations, harbour non-calcitropic phenotypes. For example, studies of the kindred with the Arg60Leu Gα_11_ mutation showed this to be associated with impaired post-natal growth, and the affected adults were significantly shorter than unaffected adult family members (height >2SD below mean of unaffected individuals) (Li *et al*. 2014). In addition, some affected members of the kindred with the Val340Met Gα_11_ mutation were found to have keratoconus, a corneal disorder (Piret *et al*. 2016).

In contrast to germline gain-of-function Gα_11_ mutations, which affect Ca^2+^_o_ homeostasis, somatic gain-of-function Gα_11_ mutations are reported to cause uveal melanoma, a primary intraocular tumour, by inducing constitutive upregulation of proliferative signalling involving extracellular signal-regulated kinases 1 and 2 (ERK1/2) (Van Raamsdonk *et al*. 2010), which are components of the MAPK signalling pathway. However, *in vitro* studies have shown that the ADH-causing Gα_11_ mutations do not have such oncogenic potential, and that these ADH-causing germline mutants phosphorylated ERK1/2 only in the presence of Ca^2+^_o_, and were thus not constitutively activating (Babinsky* et al*. 2016). Thus, the milder disturbance of signalling associated with the ADH2 mutants may provide an explanation for their occurrence as a post-natal phenotype that can be transmitted as an autosomal dominant disorder, in contrast to the uveal melanoma-associated constitutively activating G-protein mutation, Gln209Leu, which has been shown to be cytotoxic when expressed at high levels (Radhika & Dhanasekaran 2001) and is likely to be embryonically lethal if present within the germline.

An analysis of the location of ADH2-causing mutations revealed that mutated Arg60 and Arg181 residues are situated adjacent to the linker 1 and linker 2 peptides, respectively (Mannstadt *et al*. 2013, Nesbit *et al*. 2013*a*). The peptides are predicted to act as a hinge that connects the Gα_11_ GTPase domain with the helical domain, thereby allowing these two domains to form a clamshell around bound guanine nucleotides (Fig. 4). Thus, mutations affecting the Arg60 and Arg181 residues may lead to the opening of the Gα_11_ clamshell and induce G protein activation by promoting the exchange of GDP for GTP. The ADH2-causing Ser211Trp mutation is located within the region of Gα_11_, which binds to the Gβγ heterodimer, and this mutation may promote the dissociation of the Gα_11_ subunit, thus enhancing CaSR-mediated signal transduction (Mannstadt *et al*. 2013). The mutated Phe341 residue is located at the C-terminus of the Gα-subunit (Fig. 4) and forms a part of the cluster of phenylalanine residues, which likely stabilises GTP in a conformation required for its hydrolysis (Kaya* et al*. 2014, Sun* et al*. 2015), and the ADH2-causing Phe341Leu mutation is thus predicted to activate Gα_11_ by impairing the hydrolysis of GTP to GDP. In contrast to these predicted effects of the Phe341Leu mutation, the neighbouring Val340 residue is not involved in GDP/GTP exchange (Rasmussen* et al*. 2011, Sun *et al*. 2015), but instead may influence the stability of Gα-GPCR interactions (Piret *et al*. 2016).

### Use of calcimimetic and calcilytic drugs for disorders caused by Gα11 mutations

**Figure 5:**
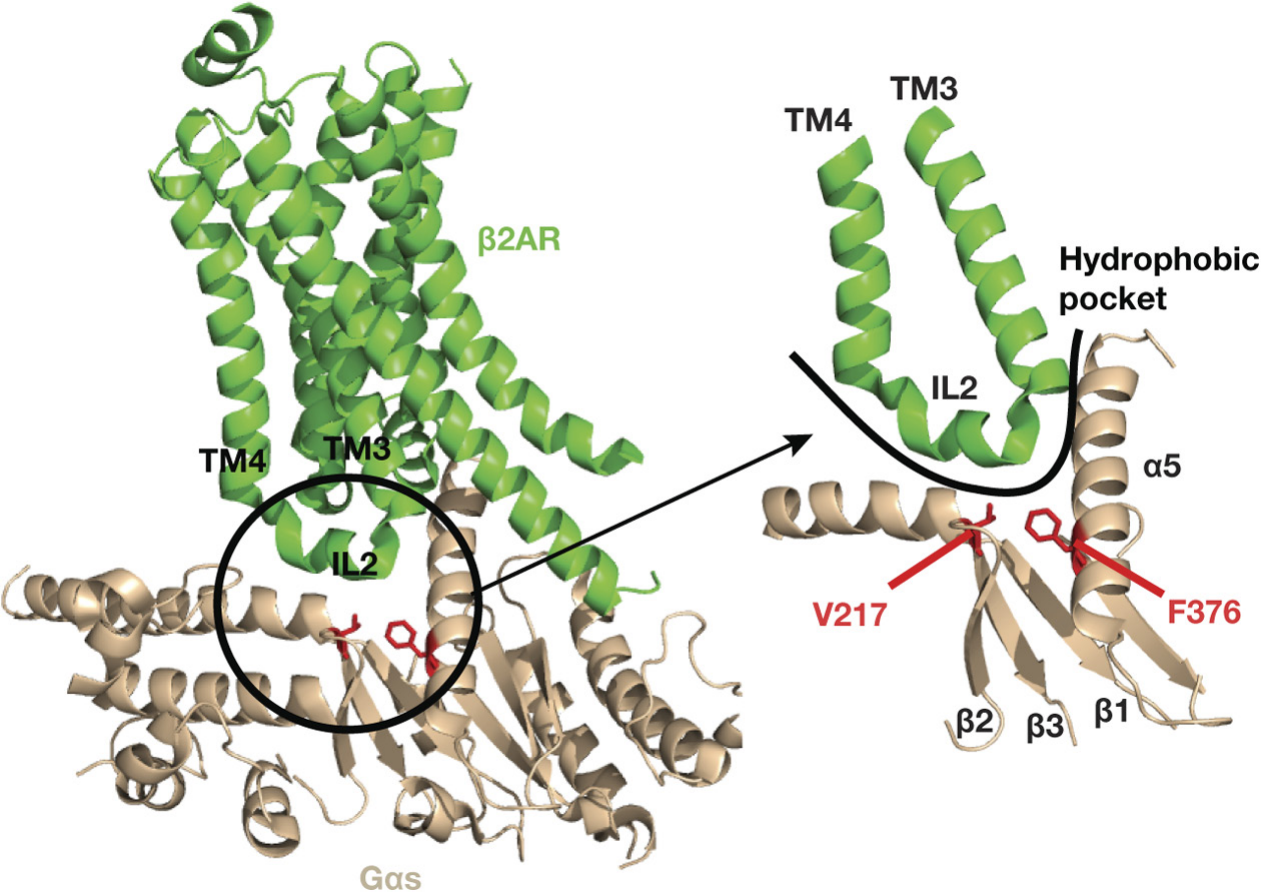
Three-dimensional model of Gαs (brown) bound to the β2-adrenergic receptor (β2AR, green) showing location of residues homologous to the Gα_11_ Ile199 and Phe341 residues, which are mutated in FHH2 and ADH2, respectively. Gαs residues homologous to the Gα_11_ Ile199 and Phe341 residues (red) are located within a hydrophobic region at the GPCR–Gα interface (black open circle). The β2AR–Gαs interface is formed by residues located within the β1 strand, hairpin loop linking the β2 and β3 strands, and the α5 helix of the Gαs protein, which interact with intracellular loop 2 (IL2) of the β2AR. The Gαs Val217 (V217) and Phe376 (F376) residues, which are homologous to the Gα_11_ Ile199 and Phe341 residues, comprise part of a hydrophobic pocket (curved line) at the Gα-subunit surface, which facilitates the docking of the β2AR IL2 with the Gαs protein (Rasmussen *et al*. 2011).

Although calcimimetic and calcilytic compounds represented targeted therapies for patients with CaSR mutations resulting in symptomatic forms of FHH1 and ADH1, it was unclear if these CaSR allosteric modulators may rectify abnormalities of the downstream Gα_11_ protein, and thus, have potential benefit for FHH2 and ADH2 patients. Recent *in vitro* studies have revealed cinacalcet and NPS-2143 to correct the loss and gain of function associated with Gα_11_ mutations leading to FHH2 and ADH2, respectively (Babinsky *et al*. 2016). Indeed, siRNA knockdown studies showed that these CaSR allosteric modulators directly influence signalling mediated by the FHH2 and ADH2 mutant Gα_11_ proteins rather than by exerting indirect effects on endogenously expressed wild-type Gα_11_ proteins (Babinsky *et al*. 2016). However, some Gα_11_ mutations (Ile199/200del and Phe341Leu) showed diminished sensitivity to cinacalcet and NPS-2143 (Babinsky *et al*. 2016) and these differences in the sensitivities of the mutants to CaSR-targeted drugs may be explained by an analysis of the crystal structure of the G protein alpha-s (Gαs) complexed with the β2-adrenergic receptor (Rasmussen *et al*. 2011). The analysis showed that residues homologous to Ile199 and Phe341, in the related Gαs protein, are located within a hydrophobic pocket at the interface between GPCR and Gα-subunit (Fig. 5). Thus, Gα_11_ mutations located at the GPCR–Gα interface may potentially influence the efficacy of CaSR allosteric modulators (Babinsky *et al*. 2016). The NPS-2143 calcilytic compound was also shown to rectify the constitutive activation caused by a uveal melanoma-associated Gα_11_ mutation, and these findings suggest a potential therapeutic role for calcilytics in the management of this intraocular tumour (Babinsky *et al*. 2016).

## Disorders associated with adaptor-related protein complex 2 sigma subunit mutations

### Familial hypocalciuric hypercalcaemia type 3 (FHH3)

**Figure 6:**
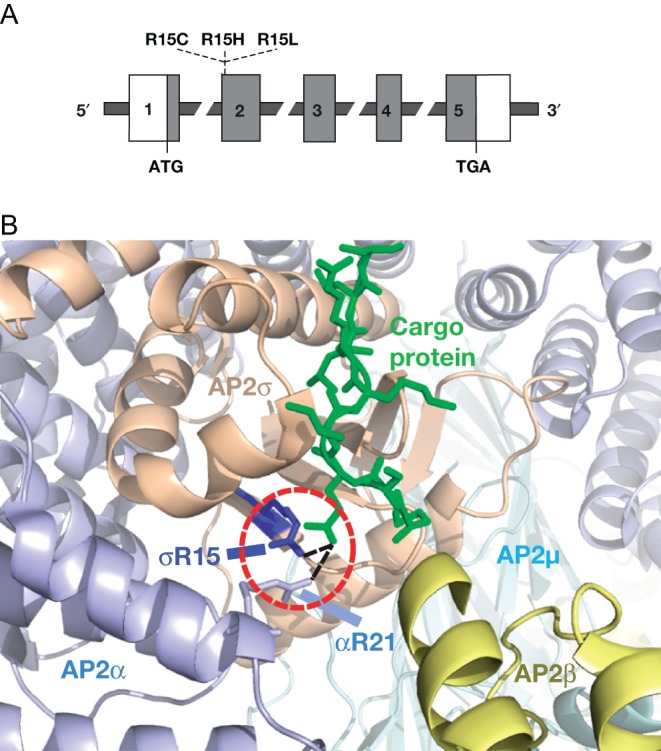
(A) Schematic representation of the genomic organisation of the *AP2S1* gene showing the location of FHH3-causing mutations. The *AP2S1* gene consists of 5 coding exons (1–5), and the start (ATG) and stop (TGA) codon are in exons 1 and 5, respectively. The 5′ portion of exon 1 and the 3′ portion of exon 5 are untranslated (open boxes). The AP2σ protein is encoded by the 3′ portion of exon 1, exons 2, 3, 4, and the 5′ portion of exon 5 (dark grey). The FHH3-causing mutations all affect the Arg15 residue and comprise Arg15Cys (R15C), Arg15His (R15H) and Arg15Leu (R15L) missense substitutions. (B) Three-dimensional model of the heterotetrameric AP2 complex, which comprises α- (purple), β- (yellow), μ- (light blue) and σ- (light brown) subunits (PDB accession number 2JKR, (Kelly *et al*. 2008)). The AP2 complex is bound to a cargo protein recognition motif (green) via key polar contacts (shown in the red dashed circle) involving the AP2σ Arg15 (R15) residue (dark blue) and Arg21 (R21) residue of the AP2α-subunit. Adapted from Nesbit MA *et al*. Nat Genet. 2013 45:93–97. Adapted, with permission, from Nesbit MA, Hannan FM, Howles SA, Reed AA, Cranston T, Thakker CE, Gregory L, Rimmer AJ, Rust N, Graham U, *et al.* (2013) Mutations in AP2S1 cause familial hypocalciuric hypercalcemia type 3. *Nature Genetics* **45** 93–97.

Genetic linkage studies of two multi-generational FHH-unrelated kindreds from Oklahoma and Northern Ireland, designated FHH_OK_ and FHH_NI_, respectively (McMurtry* et al*. 1992, Lloyd* et al*. 1999, Hannan* et al*. 2010*b*, Nesbit* et al*. 2010), mapped the disease locus, designated *FHH3*, to chromosomal 19q13.3, thereby highlighting a third genetically distinct form of FHH (FHH3, OMIM #600740) (Table 1). FHH3 was associated with elevations in plasma PTH, mild hypophosphataemia and osteomalacia in affected family members aged more than 30 years (McMurtry *et al*. 1992, Nesbit *et al*. 2010). Whole-exome capture and high-throughput sequence analysis revealed that affected individuals from the unrelated FHH_OK_ and FHH_NI_ kindreds harbour the same heterozygous germline Arg15Cys mutation of the adaptor-related protein complex 2 sigma subunit 1 (*AP2S1*) gene, which encodes the σ-subunit of the ubiquitously expressed heterotetrameric AP2 complex (Nesbit *et al*. 2013*b*). The AP2 complex is a central component of clathrin-coated vesicles and facilitates the endocytosis of plasma membrane proteins such as GPCRs (Kim & Benovic 2002). To date, *AP2S1* mutations have been reported in >60 FHH3 patients and families. All affected individuals harbour a heterozygous missense mutation affecting the Arg15 residue of the encoded AP2σ-subunit (Fig. 6) and resulting in Arg15Cys, Arg15His or Arg15Leu (Fujisawa* et al*. 2013, Nesbit *et al*. 2013*b*, Hendy* et al*. 2014, Hannan* et al*. 2015*a*, Vargas-Poussou* et al*. 2016). Crystallography studies have revealed that the Arg15 residue plays a key role in binding to membrane cargo proteins (Fig. 6) (Kelly* et al*. 2008). It is predicted that these FHH3-causing Arg15 mutations disrupt an interaction between the AP2 complex and the intracellular carboxyl terminus of the CaSR, thereby impairing endocytosis of this GPCR (Nesbit *et al*. 2013*b*). This hypothesis is supported by *in vitro* expression studies, which have demonstrated that these FHH3-causing AP2σ mutations alter CaSR cell-surface expression and impair signal transduction in a dominant-negative manner (Nesbit *et al*. 2013*b*, Hannan *et al*. 2015*a*). Thus, these studies have revealed that the AP2 endocytic complex plays a role in the regulation of Ca^2+^_o_ homeostasis.

Nucleotide substitutions affecting codon 15 of the *AP2S1* gene are predicted to result in the replacement of the wild-type Arg residue with a mutant Cys, Gly, His, Leu, Pro or Ser residue. All of these potential missense AP2σ substitutions have been demonstrated to diminish CaSR signal transduction *in vitro* (Hannan *et al*. 2015*a*). However, to date, only Arg15Cys, Arg15His and Arg15Leu AP2σ mutations have been observed in FHH3 patients (Fig. 6) (Fujisawa *et al*. 2013, Nesbit *et al*. 2013*b*, Hendy *et al*. 2014, Hannan *et al*. 2015*a*, Vargas-Poussou *et al*. 2016), and the likely cause of this mutation bias has been revealed by cellular studies. These studies have shown that the non-observed Arg15Gly, Arg15Pro and Arg15Ser AP2σ mutants impair cell growth *in vitro* (Hannan *et al*. 2015*a*). Thus, a possible explanation for the absence of the Arg15Gly, Arg15Pro and Arg15Ser AP2σ mutations in patients is that these deleterious mutations are embryonically lethal, whereas the FHH3-causing AP2σ mutations (Arg15Cys, Arg15His and Arg15Leu) are tolerated and compatible with embryonic and post-natal survival (Hannan *et al*. 2015*a*).

FHH3 may be associated with symptomatic hypercalcaemia and reduced bone mineral density, and with cognitive deficits and/or behavioural disturbances in children harbouring the Arg15Cys or Arg15Leu AP2σ mutations (Hannan *et al*. 2015*a*). FHH3 is associated with a more severe biochemical phenotype than FHH1, which is characterised by significantly higher serum calcium and magnesium concentrations and a significantly reduced fractional excretion of calcium (Hannan *et al*. 2015*a*, Vargas-Poussou *et al*. 2016). Furthermore, an analysis of adults and children with AP2σ mutations, by one study, has revealed a genotype–phenotype correlation with Arg15Leu probands having significantly higher serum calcium concentrations and presenting at a younger age, typically in childhood, compared with probands with Arg15Cys or Arg15His mutations (Hannan *et al*. 2015*a*). The absence of such a genotype–phenotype correlation in another study (Vargas-Poussou *et al*. 2016), which consisted of mainly adult FHH3 patients, suggests that the more severe hypercalcaemia associated with the Arg15Leu mutation might be age dependent.

### Use of cinacalcet for symptomatic hypercalcaemia caused by AP2σ mutations

Cinacalcet has been evaluated as a therapy for symptomatic hypercalcaemia associated with FHH3. *In vitro* studies revealed that this calcimimetic drug rectifies the significantly impaired intracellular calcium and MAPK responses associated with all three FHH3-causing AP2σ mutations (Howles* et al*. 2016). The administration of cinacalcet at a dose of 30–60 mg daily to three symptomatic FHH3 probands, each harbouring an Arg15Cys, Arg15His or Arg15Leu AP2σ mutation, led to >20% reductions in serum calcium concentrations and improved symptoms in all three FHH probands (Howles *et al*. 2016). These studies show that cinacalcet-mediated allosteric modulation of the CaSR can rectify the loss-of-function and symptomatic hypercalcaemia that are associated with the three types of FHH3-causing Arg15 AP2σ mutations. Cinacalcet has also been shown to correct the hypercalcaemia in a patient with chromosome 22q11.2 deletion syndrome who also had an Arg15Leu AP2σ mutation (Tenhola* et al*. 2015).

### Autosomal dominant hypocalcaemia type 3: the search for AP2σ mutations

Germline coding-region mutations of the *CASR* and *GNA11* genes, which enhance CaSR-mediated signal transduction, have been identified in around 70% of ADH cases (Hannan *et al*. 2012, Nesbit *et al*. 2013*a*). This raises the possibility that ADH patients who do not harbour such mutations may instead have abnormalities of the untranslated or non-coding regulatory regions of *CASR* and *GNA11* or have mutations involving other mediators of Ca^2+^_o_ homeostasis. It had been postulated that some patients with ADH might harbour AP2σ mutations, which enhance the sensitivity of CaSR-expressing cells to Ca^2+^_o_, and this putative form of ADH was designated ADH type 3 (ADH3) (Rogers* et al*. 2014). However, an analysis of the *AP2S1* gene in 19 patients and families who were considered to have ADH but did not have mutations of *CASR* and *GNA11*, or of other genes associated with isolated hypoparathyroidism, such as *PTH* or *GCMB*, failed to identify coding-region mutations or copy number variants (CNVs) (Rogers *et al*. 2014). Moreover, investigations of 10 familial cases and 50 sporadic cases of isolated hypoparathyroidism for coding-region mutations or CNVs affecting *AP2S1* did not identify any abnormalities (Lambert* et al*. 2014). Thus, these studies indicated that AP2σ mutations are unlikely to cause hypocalcaemic disorders such as ADH.

## Conclusion

The identification and characterisation of gene abnormalities underlying FHH and ADH have led to the delineation of a parathyroid and renal G protein-coupled Ca^2+^_o_-sensing mechanism, which involves the CaSR, Gα_11_ and AP2σ proteins. The CaSR is shown to play a pivotal role in the regulation of Ca^2+^_o_ concentrations, whereas the Gα_11_ protein appears to be a key mediator of downstream CaSR signal transduction, and the AP2σ protein is likely required for both CaSR signalling and trafficking. These studies have also provided new insights into the clinical phenotypes of Ca^2+^_o_-sensing disorders and revealed FHH3 to represent a distinct disorder of Ca^2+^_o_ homeostasis, which is characterised by symptomatic hypercalcaemia, low BMD and cognitive deficits. Advances have been made in the treatment of FHH and ADH, and the calcimimetic and calcilytic drugs have been shown to be of potential benefit for managing symptomatic forms of these Ca^2+^_o_-sensing disorders.

## Footnote

This review is based on the 2015 Dale Medal Lecture, presented by Prof. Rajesh Thakker at the Society for Endocrinology BES 2015, Edinburgh, UK.

## Declaration of interest

The authors declare that there is no conflict of interest that could be perceived as prejudicing the impartiality of this review.

## Funding

The work in the Academic Endocrine Unit is supported by the United Kingdom Medical Research Council (MRC) programme grants – G9825289 and G1000467, and National Institute for Health Research (NIHR) Oxford Biomedical Research Centre Programme. R V Thakker is a Wellcome Trust Investigator and NIHR Senior Investigator.

## References

[bib1] Atay Z Bereket A Haliloglu B Abali S Ozdogan T Altuncu E Canaff L Vilaca T Wong BY Cole DE 2014 Novel homozygous inactivating mutation of the calcium-sensing receptor gene (CASR) in neonatal severe hyperparathyroidism-lack of effect of cinacalcet. Bone 64 102–107. (10.1016/j.bone.2014.04.010) 24735972

[bib2] Babinsky VN Hannan FM Gorvin CM Howles SA Nesbit MA Rust N Hanyaloglu AC Hu J Spiegel AM Thakker RV 2016 Allosteric modulation of the calcium-sensing receptor rectifies signaling abnormalities associated with G-protein alpha-11 mutations causing hypercalcemic and hypocalcemic disorders. Journal of Biological Chemistry 291 10876–10885. (10.1074/jbc.M115.696401) 26994139 PMC4865932

[bib3] Bai M Quinn S Trivedi S Kifor O Pearce SH Pollak MR Krapcho K Hebert SC Brown EM 1996 Expression and characterization of inactivating and activating mutations in the human Ca2+o-sensing receptor. Journal of Biological Chemistry 271 19537–19545. (10.1074/jbc.271.32.19537) 8702647

[bib4] Breitwieser GE 2013 The calcium sensing receptor life cycle: trafficking, cell surface expression, and degradation. Best Practice & Research. Clinical Endocrinology & Metabolism 27 303–313. (10.1016/j.beem.2013.03.003) 23856261

[bib5] Breitwieser GE Gama L 2001 Calcium-sensing receptor activation induces intracellular calcium oscillations. American Journal of Physiology: Cell Physiology 280 C1412–1421. 11350736 10.1152/ajpcell.2001.280.6.C1412

[bib6] Brown EM 1991 Extracellular Ca2+ sensing, regulation of parathyroid cell function, and role of Ca2+ and other ions as extracellular (first) messengers. Physiological Reviews 71 371–411. 2006218 10.1152/physrev.1991.71.2.371

[bib7] Chattopadhyay N Brown EM 2006 Role of calcium-sensing receptor in mineral ion metabolism and inherited disorders of calcium-sensing. Molecular Genetics and Metabolism 89 189–202. (10.1016/j.ymgme.2006.07.003) 16919492

[bib8] Chikatsu N Fukumoto S Suzawa M Tanaka Y Takeuchi Y Takeda S Tamura Y Matsumoto T Fujita T 1999 An adult patient with severe hypercalcaemia and hypocalciuria due to a novel homozygous inactivating mutation of calcium-sensing receptor. Clinical Endocrinology 50 537–543. (10.1046/j.1365-2265.1999.00729.x) 10468915

[bib9] Conigrave AD Ward DT 2013 Calcium-sensing receptor (CaSR): pharmacological properties and signaling pathways. Best Practice & Research. Clinical Endocrinology & Metabolism 27 315–331. (10.1016/j.beem.2013.05.010) 23856262

[bib10] Corbetta S Lania A Filopanti M Vicentini L Ballare E Spada A 2002 Mitogen-activated protein kinase cascade in human normal and tumoral parathyroid cells. Journal of Clinical Endocrinology and Metabolism 87 2201–2205. (10.1210/jcem.87.5.8492) 11994364

[bib11] Dong B Endo I Ohnishi Y Kondo T Hasegawa T Amizuka N Kiyonari H Shioi G Abe M Fukumoto S 2015 Calcilytic ameliorates abnormalities of mutant calcium-sensing receptor (CaSR) knock-in mice mimicking autosomal dominant hypocalcemia (ADH). Journal of Bone and Mineral Research 30 1980–1993. (10.1002/jbmr.2551) 25967373

[bib12] Dore AS Okrasa K Patel JC Serrano-Vega M Bennett K Cooke RM Errey JC Jazayeri A Khan S Tehan B 2014 Structure of class C GPCR metabotropic glutamate receptor 5 transmembrane domain. Nature 511 557–562. (10.1038/nature13396) 25042998

[bib13] Festen-Spanjer B Haring CM Koster JB Mudde AH 2008 Correction of hypercalcaemia by cinacalcet in familial hypocalciuric hypercalcaemia. Clinical Endocrinology 68 324–325. (10.1111/j.1365-2265.2007.03027.x) 17803689

[bib14] Firek AF Kao PC Heath H 3rd 1991 Plasma intact parathyroid hormone (PTH) and PTH-related peptide in familial benign hypercalcemia: greater responsiveness to endogenous PTH than in primary hyperparathyroidism. Journal of Clinical Endocrinology and Metabolism 72 541–546. (10.1210/jcem-72-3-541) 1997510

[bib15] Fujisawa Y Yamaguchi R Satake E Ohtaka K Nakanishi T Ozono K Ogata T 2013 Identification of AP2S1 mutation and effects of low calcium formula in an infant with hypercalcemia and hypercalciuria. Journal of Clinical Endocrinology and Metabolism 98 E2022–E2027. (10.1210/jc.2013-2571) 24081735

[bib16] Gannon AW Monk HM Levine MA 2014 Cinacalcet monotherapy in neonatal severe hyperparathyroidism: a case study and review. Journal of Clinical Endocrinology and Metabolism 99 7–11. (10.1210/jc.2013-2834) 24203066 PMC3879678

[bib17] Gorvin CM Cranston T Hannan FM Rust N Qureshi A Nesbit MA Thakker RV 2016 G-Protein subunit-alpha11 loss-of-function mutation, Thr54Met, causing familial hypocalciuric hypercalcemia type 2 (FHH2). Journal of Bone and Mineral Research 31 1200–1206. (10.1002/jbmr.2778) 26729423 PMC4949650

[bib18] Grant MP Stepanchick A Cavanaugh A Breitwieser GE 2011 Agonist-driven maturation and plasma membrane insertion of calcium-sensing receptors dynamically control signal amplitude. Science Signaling 4 ra78 (10.1126/scisignal.2002208) 22114145

[bib19] Grant MP Stepanchick A Breitwieser GE 2012 Calcium signaling regulates trafficking of familial hypocalciuric hypercalcemia (FHH) mutants of the calcium sensing receptor. Molecular Endocrinology 26 2081–2091. (10.1210/me.2012-1232) 23077345 PMC5416950

[bib20] Hannan FM Nesbit MA Christie PT Lissens W Van der Schueren B Bex M Bouillon R Thakker RV 2010a A homozygous inactivating calcium-sensing receptor mutation, Pro339Thr, is associated with isolated primary hyperparathyroidism: correlation between location of mutations and severity of hypercalcaemia. Clinical Endocrinology 73 715–722. (10.1111/j.1365-2265.2010.03870.x) 20846291

[bib21] Hannan FM Nesbit MA Turner JJ Stacey JM Cianferotti L Christie PT Conigrave AD Whyte MP Thakker RV 2010b Comparison of human chromosome 19q13 and syntenic region on mouse chromosome 7 reveals absence, in man, of 11.6 Mb containing four mouse calcium-sensing receptor-related sequences: relevance to familial benign hypocalciuric hypercalcaemia type 3. European Journal of Human Genetics 18 442–447. (10.1038/ejhg.2009.161) 19809483 PMC2842244

[bib22] Hannan FM Nesbit MA Zhang C Cranston T Curley AJ Harding B Fratter C Rust N Christie PT Turner JJ 2012 Identification of 70 calcium-sensing receptor mutations in hyper- and hypo-calcaemic patients: evidence for clustering of extracellular domain mutations at calcium-binding sites. Human Molecular Genetics 21 2768–2778. (10.1093/hmg/dds105) 22422767

[bib23] Hannan FM Thakker RV 2013 Calcium-sensing receptor (CaSR) mutations and disorders of calcium, electrolyte and water metabolism. Best Practice & Research. Clinical Endocrinology & Metabolism 27 359–371. (10.1016/j.beem.2013.04.007) 23856265

[bib24] Hannan FM Howles SA Rogers A Cranston T Gorvin CM Babinsky VN Reed AA Thakker CE Bockenhauer D Brown RS 2015a Adaptor protein-2 sigma subunit mutations causing familial hypocalciuric hypercalcaemia type 3 (FHH3) demonstrate genotype-phenotype correlations, codon bias and dominant-negative effects. Human Molecular Genetics 24 5079–5092. (10.1093/hmg/ddv226) 26082470 PMC4550820

[bib25] Hannan FM Walls GV Babinsky VN Nesbit MA Kallay E Hough TA Fraser WD Cox RD Hu J Spiegel AM 2015b The calcilytic agent NPS 2143 rectifies hypocalcemia in a mouse model with an activating calcium-sensing receptor (CaSR) mutation: relevance to autosomal dominant hypocalcemia type 1 (ADH1). Endocrinology 156 3114–3121. (10.1210/en.2015-1269) 26052899 PMC4541614

[bib26] Heath H 3rd Jackson CE Otterud B Leppert MF 1993 Genetic linkage analysis in familial benign (hypocalciuric) hypercalcemia: evidence for locus heterogeneity. American Journal of Human Genetics 53 193–200. 8317484 PMC1682230

[bib27] Hendy GN Canaff L Newfield RS Tripto-Shkolnik L Wong BY Lee BS Cole DE 2014 Codon Arg15 Mutations of the AP2S1 gene: common occurrence in familial hypocalciuric hypercalcemia cases negative for calcium-sensing receptor (CASR) mutations. Journal of Clinical Endocrinology and Metabolism 99 E1311–E1315. (10.1210/jc.2014-1120) 24731014

[bib28] Hofer AM Brown EM 2003 Extracellular calcium sensing and signalling. Nature Reviews. Molecular Cell Biology 4 530–538. (10.1038/nrm1154) 12838336

[bib29] Hough TA Bogani D Cheeseman MT Favor J Nesbit MA Thakker RV Lyon MF 2004 Activating calcium-sensing receptor mutation in the mouse is associated with cataracts and ectopic calcification. PNAS 101 13566–13571. (10.1073/pnas.0405516101) 15347804 PMC518795

[bib30] Howles SA Hannan FM Babinsky VN Rogers A Gorvin CM Rust N Nesbit MA Thakker RV Richardson T McKenna MJ 2016 Cinacalcet for symptomatic hypercalcemia caused by AP2S1 mutations. New England Journal of Medicine 374 1396–1398. (10.1056/NEJMc1511646) 27050234 PMC4972445

[bib31] Hu J Spiegel AM 2007 Structure and function of the human calcium-sensing receptor: insights from natural and engineered mutations and allosteric modulators. Journal of Cellular and Molecular Medicine 11 908–922. (10.1111/j.1582-4934.2007.00096.x) 17979873 PMC4401263

[bib32] Hu J McLarnon SJ Mora S Jiang J Thomas C Jacobson KA Spiegel AM 2005 A region in the seven-transmembrane domain of the human Ca2+ receptor critical for response to Ca2+. Journal of Biological Chemistry 280 5113–5120. (10.1074/jbc.M413403200) 15591042

[bib33] Huang Y Breitwieser GE 2007 Rescue of calcium-sensing receptor mutants by allosteric modulators reveals a conformational checkpoint in receptor biogenesis. Journal of Biological Chemistry 282 9517–9525. (10.1074/jbc.M609045200) 17284438

[bib34] Huang Y Zhou Y Yang W Butters R Lee HW Li S Castiblanco A Brown EM Yang JJ 2007 Identification and dissection of Ca(2+)-binding sites in the extracellular domain of Ca(2+)-sensing receptor. Journal of Biological Chemistry 282 19000–19010. (10.1074/jbc.M701096200) 17478419 PMC2867057

[bib35] Huang Y Zhou Y Castiblanco A Yang W Brown EM Yang JJ 2009 Multiple Ca(2+)-binding sites in the extracellular domain of the Ca(2+)-sensing receptor corresponding to cooperative Ca(2+) response. Biochemistry 48 388–398. (10.1021/bi8014604) 19102677 PMC2627791

[bib36] Jensen AA Spalding TA Burstein ES Sheppard PO O’Hara PJ Brann MR Krogsgaard-Larsen P Brauner-Osborne H 2000 Functional importance of the Ala(116)-Pro(136) region in the calcium-sensing receptor. Constitutive activity and inverse agonism in a family C G-protein-coupled receptor. Journal of Biological Chemistry 275 29547–29555. (10.1074/jbc.M910023199) 10835431

[bib37] Katritch V Cherezov V Stevens RC 2013 Structure-function of the G protein-coupled receptor superfamily. Annual Review of Pharmacology and Toxicology 53 531–556. (10.1146/annurev-pharmtox-032112-135923) PMC3540149 23140243

[bib38] Kaya AI Lokits AD Gilbert JA Iverson TM Meiler J Hamm HE 2014 A conserved phenylalanine as a relay between the alpha5 helix and the GDP binding region of heterotrimeric Gi protein alpha subunit. Journal of Biological Chemistry 289 24475–24487. (10.1074/jbc.M114.572875) 25037222 PMC4148873

[bib39] Kelly BT McCoy AJ Spate K Miller SE Evans PR Honing S Owen DJ 2008 A structural explanation for the binding of endocytic dileucine motifs by the AP2 complex. Nature 456 976–979. (10.1038/nature07422) 19140243 PMC4340503

[bib40] Kim YM Benovic JL 2002 Differential roles of arrestin-2 interaction with clathrin and adaptor protein 2 in G protein-coupled receptor trafficking. Journal of Biological Chemistry 277 30760–30768. (10.1074/jbc.M204528200) 12070169

[bib41] Kinoshita Y Hori M Taguchi M Watanabe S Fukumoto S 2014 Functional activities of mutant calcium-sensing receptors determine clinical presentations in patients with autosomal dominant hypocalcemia. Journal of Clinical Endocrinology and Metabolism 99 E363–E368. (10.1210/jc.2013-3430) 24297799

[bib42] Lambert AS Grybek V Francou B Esterle L Bertrand G Bouligand J Guiochon-Mantel A Hieronimus S Voitel D Soskin S 2014 Analysis of AP2S1, a calcium-sensing receptor regulator, in familial and sporadic isolated hypoparathyroidism. Journal of Clinical Endocrinology and Metabolism 99 E469–E473. (10.1210/jc.2013-2572) 24423332

[bib43] Leach K Wen A Davey AE Sexton PM Conigrave AD Christopoulos A 2012 Identification of molecular phenotypes and biased signaling induced by naturally occurring mutations of the human calcium-sensing receptor. Endocrinology 153 4304–4316. (10.1210/en.2012-1449) 22798347

[bib44] Leach K Wen A Cook AE Sexton PM Conigrave AD Christopoulos A 2013 Impact of clinically relevant mutations on the pharmacoregulation and signaling bias of the calcium-sensing receptor by positive and negative allosteric modulators. Endocrinology 154 1105–1116. (10.1210/en.2012-1887) 23372019

[bib45] Leach K Gregory KJ Kufareva I Khajehali E Cook AE Abagyan R Conigrave AD Sexton PM Christopoulos A 2016 Towards a structural understanding of allosteric drugs at the human calcium-sensing receptor. Cell Research 26 574–592. (10.1038/cr.2016.36) 27002221 PMC4856764

[bib46] Letz S Rus R Haag C Dorr HG Schnabel D Mohlig M Schulze E Frank-Raue K Raue F Mayr B 2010 Novel activating mutations of the calcium-sensing receptor: the calcilytic NPS-2143 mitigates excessive signal transduction of mutant receptors. Journal of Clinical Endocrinology and Metabolism 95 E229–E233. (10.1210/jc.2010-0651) 20668040

[bib47] Letz S Haag C Schulze E Frank-Raue K Raue F Hofner B Mayr B Schofl C 2014 Amino alcohol- (NPS-2143) and quinazolinone-derived calcilytics (ATF936 and AXT914) differentially mitigate excessive signalling of calcium-sensing receptor mutants causing Bartter syndrome Type 5 and autosomal dominant hypocalcemia. PLoS ONE 9 e115178 (10.1371/journal.pone.0115178) 25506941 PMC4266668

[bib48] Li D Opas EE Tuluc F Metzger DL Hou C Hakonarson H Levine MA 2014 Autosomal dominant hypoparathyroidism caused by germline mutation in GNA11: phenotypic and molecular characterization. Journal of Clinical Endocrinology and Metabolism 99 E1774–E1783. (10.1210/jc.2014-1029) 24823460 PMC4154081

[bib49] Lia-Baldini AS Magdelaine C Nizou A Airault C Salles JP Moulin P Delemer B Aitouares M Funalot B Sturtz F 2013 Two novel mutations of the calcium-sensing receptor gene affecting the same amino acid position lead to opposite phenotypes and reveal the importance of p.N802 on receptor activity. European Journal of Endocrinology 168 K27–K34. (10.1530/EJE-12-0714) 23169696

[bib50] Lloyd SE Pannett AA Dixon PH Whyte MP Thakker RV 1999 Localization of familial benign hypercalcemia, Oklahoma variant (FBHOk), to chromosome 19q13. American Journal of Human Genetics 64 189–195. (10.1086/302202) 9915958 PMC1377717

[bib51] Loupy A Ramakrishnan SK Wootla B Chambrey R de la Faille R Bourgeois S Bruneval P Mandet C Christensen EI Faure H 2012 PTH-independent regulation of blood calcium concentration by the calcium-sensing receptor. Journal of Clinical Investigation 122 3355–3367. (10.1172/JCI57407) 22886306 PMC3428075

[bib52] Mannstadt M Harris M Bravenboer B Chitturi S Dreijerink KM Lambright DG Lim ET Daly MJ Gabriel S Juppner H 2013 Germline mutations affecting Galpha11 in hypoparathyroidism. New England Journal of Medicine 368 2532–2534. (10.1056/NEJMc1300278) 23802536 PMC3750735

[bib53] Marx SJ 2015 Letter to the editor: Distinguishing typical primary hyperparathyroidism from familial hypocalciuric hypercalcemia by using an index of urinary calcium. Journal of Clinical Endocrinology and Metabolism 100 L29–L30. (10.1210/jc.2014-4221) 25658165 PMC5393510

[bib54] McMurtry CT Schranck FW Walkenhorst DA Murphy WA Kocher DB Teitelbaum SL Rupich RC Whyte MP 1992 Significant developmental elevation in serum parathyroid hormone levels in a large kindred with familial benign (hypocalciuric) hypercalcemia. American Journal of Medicine 93 247–258. (10.1016/0002-9343(92)90229-5) 1524075

[bib55] Miyashiro K Kunii I Manna TD de Menezes Filho HC Damiani D Setian N Hauache OM 2004 Severe hypercalcemia in a 9-year-old Brazilian girl due to a novel inactivating mutation of the calcium-sensing receptor. Journal of Clinical Endocrinology and Metabolism 89 5936–5941. (10.1210/jc.2004-1046) 15579740

[bib56] Murphy H Patrick J Baez-Irizarry E Lacassie Y Gomez R Vargas A Barkemeyer B Kanotra S Zambrano RM 2016 Neonatal severe hyperparathyroidism caused by homozygous mutation in CASR: a rare cause of life-threatening hypercalcemia. European Journal of Medical Genetics 59 227–231. (10.1016/j.ejmg.2016.02.001) 26855056

[bib57] Nemeth EF Goodman WG 2016 Calcimimetic and calcilytic drugs: feats, flops, and futures. Calcified Tissue International 98 341–358. (10.1007/s00223-015-0052-z) 26319799

[bib58] Nemeth EF Shoback D 2013 Calcimimetic and calcilytic drugs for treating bone and mineral-related disorders. Best Practice & Research. Clinical Endocrinology & Metabolism 27 373–384. (10.1016/j.beem.2013.02.008) 23856266

[bib59] Nemeth EF Heaton WH Miller M Fox J Balandrin MF Van Wagenen BC Colloton M Karbon W Scherrer J Shatzen E 2004 Pharmacodynamics of the type II calcimimetic compound cinacalcet HCl. Journal of Pharmacology and Experimental Therapeutics 308 627–635. (10.1124/jpet.103.057273) 14593085

[60] Nesbit MA Hannan FM Graham U Whyte MP Morrison PJ Hunter SJ Thakker RV 2010 Identification of a second kindred with familial hypocalciuric hypercalcemia type 3 (FHH3) narrows localization to a <3.5 megabase pair region on chromosome 19q13.3. Journal of Clinical Endocrinology and Metabolism 95 1947–1954. 20133464 10.1210/jc.2009-2152

[bib61] Nesbit MA Hannan FM Howles SA Babinsky VN Head RA Cranston T Rust N Hobbs MR Heath H 3rd Thakker RV 2013a Mutations affecting G-protein subunit alpha11 in hypercalcemia and hypocalcemia. New England Journal of Medicine 368 2476–2486. (10.1056/NEJMoa1300253) 23802516 PMC3773604

[bib62] Nesbit MA Hannan FM Howles SA Reed AA Cranston T Thakker CE Gregory L Rimmer AJ Rust N Graham U 2013b Mutations in AP2S1 cause familial hypocalciuric hypercalcemia type 3. Nature Genetics 45 93–97. (10.1038/ng.2492) 23222959 PMC3605788

[bib63] Nishimura A Kitano K Takasaki J Taniguchi M Mizuno N Tago K Hakoshima T Itoh H 2010 Structural basis for the specific inhibition of heterotrimeric Gq protein by a small molecule. PNAS 107 13666–13671. (10.1073/pnas.1003553107) 20639466 PMC2922266

[bib64] Oldham WM Hamm HE 2008 Heterotrimeric G protein activation by G-protein-coupled receptors. Nature Reviews. Molecular Cell Biology 9 60–71. (10.1038/nrm2299) 18043707

[bib65] Pearce SH Bai M Quinn SJ Kifor O Brown EM Thakker RV 1996a Functional characterization of calcium-sensing receptor mutations expressed in human embryonic kidney cells. Journal of Clinical Investigation 98 1860–1866. (10.1172/JCI118987) 8878438 PMC507626

[66] Pearce SH Williamson C Kifor O Bai M Coulthard MG Davies M Lewis-Barned N McCredie D Powell H Kendall-Taylor P 1996b A familial syndrome of hypocalcemia with hypercalciuria due to mutations in the calcium-sensing receptor. New England Journal of Medicine 335 1115–1122. (10.1056/NEJM199610103351505) 8813042

[bib67] Pearce SH Wooding C Davies M Tollefsen SE Whyte MP Thakker RV 1996c Calcium-sensing receptor mutations in familial hypocalciuric hypercalcaemia with recurrent pancreatitis. Clinical Endocrinology 45 675–680. (10.1046/j.1365-2265.1996.750891.x) 9039332

[bib68] Piret SE Gorvin CM Pagnamenta AT Howles SA Cranston T Rust N Nesbit MA Glaser B Taylor JC Buchs AE 2016 Identification of a G-protein subunit-alpha11 gain-of-function mutation, Val340Met, in a family with autosomal dominant hypocalcemia type 2 (ADH2). Journal of Bone and Mineral Research 31 1207–1214. (10.1002/jbmr.2797) 26818911 PMC4915495

[bib69] Pollak MR Brown EM Chou YH Hebert SC Marx SJ Steinmann B Levi T Seidman CE Seidman JG 1993 Mutations in the human Ca(2+)-sensing receptor gene cause familial hypocalciuric hypercalcemia and neonatal severe hyperparathyroidism. Cell 75 1297–1303. (10.1016/0092-8674(93)90617-Y) 7916660

[bib70] Pollak MR Brown EM Estep HL McLaine PN Kifor O Park J Hebert SC Seidman CE Seidman JG 1994 Autosomal dominant hypocalcaemia caused by a Ca(2+)-sensing receptor gene mutation. Nature Genetics 8 303–307. (10.1038/ng1194-303) 7874174

[bib71] Radhika V Dhanasekaran N 2001 Transforming G proteins. Oncogene 20 1607–1614. (10.1038/sj.onc.1204274) 11313908

[bib72] Ramnitz M Gafni RI Brillante B Guthrie L Gash D Gelb J Krusinska E Brennan SC Bin Khayat ME Ward DT 2015 Treatment of autosomal dominant hypocalcemia with the calcilytic NPSP795. Journal of Bone and Mineral Research 30 (Suppl 1). 10.1002/jbmr.3747 PMC6744344 31063613

[bib73] Rasmussen SG DeVree BT Zou Y Kruse AC Chung KY Kobilka TS Thian FS Chae PS Pardon E Calinski D 2011 Crystal structure of the beta2 adrenergic receptor-Gs protein complex. Nature 477 549–555. (10.1038/nature10361) 21772288 PMC3184188

[bib74] Regard JB Sato IT Coughlin SR 2008 Anatomical profiling of G protein-coupled receptor expression. Cell 135 561–571. (10.1016/j.cell.2008.08.040) 18984166 PMC2590943

[bib75] Riccardi D Valenti G 2016 Localization and function of the renal calcium-sensing receptor. Nature Reviews Nephrology 12 414–425. (10.1038/nrneph.2016.59) 27157444

[bib76] Rogers A Nesbit MA Hannan FM Howles SA Gorvin CM Cranston T Allgrove J Bevan JS Bano G Brain C 2014 Mutational analysis of the adaptor protein 2 sigma subunit (AP2S1) gene: search for autosomal dominant hypocalcemia type 3 (ADH3). Journal of Clinical Endocrinology and Metabolism 99 E1300–E1305. (10.1210/jc.2013-3909) 24708097 PMC4447854

[bib77] Rus R Haag C Bumke-Vogt C Bahr V Mayr B Mohlig M Schulze E Frank-Raue K Raue F Schofl C 2008 Novel inactivating mutations of the calcium-sensing receptor: the calcimimetic NPS R-568 improves signal transduction of mutant receptors. Journal of Clinical Endocrinology and Metabolism 93 4797–4803. (10.1210/jc.2008-1076) 18796518

[bib78] Sun D Flock T Deupi X Maeda S Matkovic M Mendieta S Mayer D Dawson RJ Schertler GF Babu MM 2015 Probing Galphai1 protein activation at single-amino acid resolution. Nature Structural & Molecular Biology 22 686–694. (10.1038/nsmb.3070) PMC4876908 26258638

[bib79] Tenhola S Hendy GN Valta H Canaff L Lee BS Wong BY Valimaki MJ Cole DE Makitie O 2015 Cinacalcet treatment in an adolescent with concurrent 22q11.2 deletion syndrome and FHH3 caused by AP2S1 mutation. Journal of Clinical Endocrinology and Metabolism 100 2515–2518. (10.1210/jc.2015-1518) 25993639

[bib80] Thakker RV Bringhurst FR Juppner H 2016 Endocrinology: Adult & Pediatric. Philadelphia, PA, USA: Saunders/Elsevier.

[bib81] Theman TA Collins MT Dempster DW Zhou H Reynolds JC Brahim JS Roschger P Klaushofer K Winer KK 2009 PTH(1-34) replacement therapy in a child with hypoparathyroidism caused by a sporadic calcium receptor mutation. Journal of Bone and Mineral Research 24 964–973. (10.1359/jbmr.081233) 19063686 PMC2672210

[bib82] Timmers HJ Karperien M Hamdy NA de Boer H Hermus AR 2006 Normalization of serum calcium by cinacalcet in a patient with hypercalcaemia due to a de novo inactivating mutation of the calcium-sensing receptor. Journal of Internal Medicine 260 177–182. (10.1111/j.1365-2796.2006.01684.x) 16882283

[bib83] Van Raamsdonk CD Griewank KG Crosby MB Garrido MC Vemula S Wiesner T Obenauf AC Wackernagel W Green G Bouvier N 2010 Mutations in GNA11 in uveal melanoma. New England Journal of Medicine 363 2191–2199. (10.1056/NEJMoa1000584) 21083380 PMC3107972

[bib84] Vargas-Poussou R Huang C Hulin P Houillier P Jeunemaitre X Paillard M Planelles G Dechaux M Miller RT Antignac C 2002 Functional characterization of a calcium-sensing receptor mutation in severe autosomal dominant hypocalcemia with a Bartter-like syndrome. Journal of the American Society of Nephrology 13 2259–2266. (10.1097/01.ASN.0000025781.16723.68) 12191970

[bib85] Vargas-Poussou R Mansour-Hendili L Baron S Bertocchio JP Travers C Simian C Treard C Baudouin V Beltran S Broux F 2016 Familial hypocalciuric hypercalcemia types 1 and 3 and primary hyperparathyroidism: similarities and differences. Journal of Clinical Endocrinology and Metabolism 101 2185–2195. (10.1210/jc.2015-3442) 26963950

[bib86] Varrault A Pena MS Goldsmith PK Mithal A Brown EM Spiegel AM 1995 Expression of G protein alpha-subunits in bovine parathyroid. Endocrinology 136 4390–4396. (10.1210/endo.136.10.7664659) 7664659

[bib87] Volpe A Guerriero A Marchetta A Caramaschi P Furlani L 2009 Familial hypocalciuric hypercalcemia revealed by chondrocalcinosis. Joint Bone Spine 76 708–710. (10.1016/j.jbspin.2009.02.001) 19467900

[bib88] Waller S Kurzawinski T Spitz L Thakker R Cranston T Pearce S Cheetham T van’t Hoff WG 2004 Neonatal severe hyperparathyroidism: genotype/phenotype correlation and the use of pamidronate as rescue therapy. European Journal of Pediatrics 163 589–594. (10.1007/s00431-004-1491-0) 15241688

[bib89] Watanabe S Fukumoto S Chang H Takeuchi Y Hasegawa Y Okazaki R Chikatsu N Fujita T 2002 Association between activating mutations of calcium-sensing receptor and Bartter’s syndrome. Lancet 360 692–694. (10.1016/S0140-6736(02)09842-2) 12241879

[bib90] Wettschureck N Lee E Libutti SK Offermanns S Robey PG Spiegel AM 2007 Parathyroid-specific double knockout of Gq and G11 alpha-subunits leads to a phenotype resembling germline knockout of the extracellular Ca2+ -sensing receptor. Molecular Endocrinology 21 274–280. (10.1210/me.2006-0110) 16988000

[bib91] White E McKenna J Cavanaugh A Breitwieser GE 2009 Pharmacochaperone-mediated rescue of calcium-sensing receptor loss-of-function mutants. Molecular Endocrinology 23 1115–1123. (10.1210/me.2009-0041) 19389809 PMC2703600

[bib92] Wilhelm-Bals A Parvex P Magdelaine C Girardin E 2012 Successful use of bisphosphonate and calcimimetic in neonatal severe primary hyperparathyroidism. Pediatrics 129 e812–e816. (10.1542/peds.2011-0128) 22331334

[bib93] Zhang C Mulpuri N Hannan FM Nesbit MA Thakker RV Hamelberg D Brown EM Yang JJ 2014 Role of Ca2+ and L-Phe in regulating functional cooperativity of disease-associated “toggle” calcium-sensing receptor mutations. PLoS ONE 9 e113622 (10.1371/journal.pone.0113622) 25420019 PMC4242666

[bib94] Zhang C Zhang T Zou J Miller CL Gorkhali R Yang J-Y Schilmiller A Wang S Huang K Brown EM 2016 Structural basis for regulation of human calcium-sensing receptor by magnesium ions and an unexpected tryptophan derivative co-agonist. Science Advances 2 e1600241 (10.1126/sciadv.1600241) 27386547 PMC4928972

